# Cognitive Flexibility Mediates the Associations Between Perceived Stress, Social Camouflaging and Mental Health Challenges in Autistic Adults

**DOI:** 10.1002/aur.70061

**Published:** 2025-06-03

**Authors:** Matthew J. Hollocks, Goldie A. McQuaid, Nancy R. Lee, Gregory L. Wallace

**Affiliations:** ^1^ Department of Child & Adolescent Psychiatry King's College London London UK; ^2^ Department of Psychology George Mason University Fairfax Virginia USA; ^3^ Department of Psychological and Brain Sciences Drexel University Philadelphia Pennsylvania USA; ^4^ Department of Speech, Language, and Hearing Sciences The George Washington University Washington District of Columbia USA

## Abstract

Autistic people are at an elevated risk of experiencing co‐occurring anxiety and depression. The contributors to this are likely multifaceted and complex and remain poorly understood. Cognitive flexibility, social camouflaging, and perceived stress provide useful indices of the interacting neurocognitive, behavioral, and environmental factors that have been associated with anxiety and depression in autistic individuals. Here, we test if cognitive flexibility, as the factor most closely related to individual differences in thinking styles, mediates the relationships between social camouflaging, perceived stress, and anxiety/depression. This study included 806 autistic individuals aged between 18 and 83 years (Mean age = 40.2), recruited through the Research Match service of the Simons Powering Autism Research (SPARK) participant registry. Participants completed an online battery of questionnaires measuring cognitive and social flexibility, social camouflaging, perceived stress, anxiety, and depression. Parallel mediation analyses were used to test the mediating effect of cognitive and social flexibility. Across separate parallel mediation analyses, cognitive flexibility was found to significantly mediate the relationships between both social camouflaging and perceived stress with anxiety and depression. This was contrasted with social flexibility, which showed a lower magnitude mediating effect for perceived stress and no mediating effect of social camouflaging. Cognitive flexibility plays an important mediating role between the impact of both perceived stress and social camouflaging on greater symptoms of both anxiety and depression in autistic adults.

**Summary**

- Camouflaging refers to ways people “hide” being autistic.
- Camouflaging is associated with anxiety and depression.
- Perceived stress, or how stressful a person sees their life, is also associated with anxiety and depression.
- We looked at whether flexibility, or the ability to think and behave flexibly, is associated with relationships between camouflaging and perceived stress with anxiety and depression.
- Specifically, we looked at whether the relationship between perceived stress and camouflaging with anxiety and depression symptoms is explained by flexibility.
- We found that flexibility explained associations of both perceived stress and social camouflaging with anxiety and depression.
- Future research should look at flexible thinking to help autistic people's mental health.

## 1 Introduction

Autistic people are at an elevated risk of experiencing co‐occurring anxiety and depression, with a lifetime prevalence in adulthood of 42% and 37%, respectively (Hollocks et al. 2019). This is much higher than would be expected when compared to individuals who do not have a diagnosis of autism, where the lifetime prevalence for anxiety is around 34%, and 30% for depression (Kessler et al. 2012). The reason for this increased risk remains unclear, although it is likely to be related to a combination of thinking differences associated with autism, social and environmental risk factors, and their interaction. In terms of thinking differences, challenges with cognitive flexibility (Hollocks et al. 2022; Lei et al. 2022) and an associated propensity for intolerance of uncertainty (Vasa et al. 2018) are likely key contributors. These thinking differences likely interact with environments that are stress‐inducing for autistic individuals (due to sensory or other processing differences or social demands), resulting in behavioral strategies through which stressful or anxiety‐provoking environments can be managed (Lei et al. 2022). Environmental factors may include exposure to bullying and adverse life events (Hartley et al. 2024), stress associated with belonging to a neurominority group (Botha and Frost 2020), and negative systemic messaging about being autistic across various settings over time (e.g., home, schools, workplace, etc.).

Cognitive flexibility is a core domain of executive functioning (Miyake and Friedman 2012) which encapsulates an individual's readiness to selectively switch between cognitive processes to generate a context‐appropriate behavioral response (Uddin 2021) and incorporates a range of skills including attentional and set shifting (Dajani and Uddin 2015) as well as generativity and reward sensitivity (Hauser et al. 2015). Autistic people experience lower levels of cognitive flexibility when compared to those without an autism diagnosis (Bertollo et al. 2020), and this has been associated directly with increased symptoms of both anxiety and depression (Hollocks et al. 2022; Lawson et al. 2015; Ozsivadjian et al. 2021; Wallace et al. 2016). In a sample of autistic adults, cognitive flexibility difficulties have been associated with both higher levels of stress and elevated anxiety and depression (Lee et al. 2022). Whether reduced cognitive flexibility should be considered a direct risk factor for co‐occurring mental health difficulties in autism, or rather is better thought of as a mediator of other distinct processes (e.g., exposure to environmental stressors), has yet to be established. Repetitive or perseverative patterns of thinking have been linked with the severity of anxiety and depression, their degree of co‐occurrence, as well as the persistence and relapse of symptoms, in those without autism (Spinhoven et al. 2018). Challenges with flexible thinking and executive functions have been shown to be associated with social functioning (Kenworthy et al. 2014; social flexibility), a key area of difficulty for autistic people and one that may contribute to mental health difficulties. These findings suggest that supports which target cognitive flexibility could be an important consideration as a future treatment innovation, particularly, should flexibility act as a mediating factor between other risk factors/behaviors and suboptimal psychiatric outcomes in autistic people.

Carter Leno et al. (2022) have suggested that in autistic people, cognitive flexibility may mediate the impact of exposure to stressful life events in childhood on later mental health. Higher endorsement of stress has previously been linked with lower performance on general measurements of executive functioning, which in turn is correlated with poorer mental health (Demetriou et al. 2021). An individual's appraisal of how stressful their day‐to‐day life is, and their resources to manage this, is referred to as perceived stress. Autistic adults report elevated levels of perceived stress relative to those without a diagnosis (Bishop‐Fitzpatrick et al. 2017; McQuaid, Weiss, et al. 2022), and increased endorsement of stress is associated with more symptoms of anxiety and depression (McQuaid et al. 2024). In the allistic/non‐autistic adult population, decreased cognitive flexibility has been associated with higher levels of stress (Goldfarb et al. 2017), and a subsequent risk for the development of psychiatric conditions (Stange et al. 2016). However, the specific role cognitive flexibility plays in mediating the association between perceived stress and mental health has yet to be investigated in autistic adults.

Another factor that has been suggested to be associated with poor mental health in autistic individuals is social camouflaging (Hull et al. 2017, 2021; McQuaid et al. 2024). Social camouflaging includes engagement in compensatory strategies which include the conscious or subconscious masking of social and communication difficulties associated with autism. These include masking strategies that allow one to present a “nonautistic” persona and assimilation strategies that can be used to “fit in” to social situations (Hull et al. 2019). Social camouflaging behaviors have been associated with exhaustion, burnout and more mental health challenges in autistic adults (Beck et al. 2020; Cage and Troxell‐Whitman 2019; Hull et al. 2019, 2021; Lai et al. 2019), suggesting that social camouflaging can be linked to considerable costs. Recently, Lei et al. (2022) demonstrated in a sample of autistic and nonautistic adolescents with social anxiety that elevated levels of anxiety are associated with masking behaviors, suggesting that masking may be a form of anxiety related safety behavior, similar to those described in cognitive behavioral models of social phobia. This has implications for not only treatment, but also the potential role of reduced cognitive flexibility as one mechanism through which social camouflaging may be associated with anxiety and depression. Set shifting, a component of cognitive flexibility has been shown to be a key factor in cognitive re‐structuring (taking a different perspective on your thoughts and experiences) in those with social anxiety (Holder et al. 2021). Whilst for some individuals there are likely some positive benefits of social camouflaging (Bradley et al. 2021), reduced cognitive flexibility may increase the cognitive burden we know is associated with social camouflaging (Raymaker et al. 2020) and through reduced flexibility in responding to stressful environments contribute to mental health challenges.

Having established that perceived stress and social camouflaging are both significantly associated with elevated anxiety and depression in autistic individuals, it is now important to investigate how these environmental and behavioral factors may interact with cognitive flexibility as one potentially modifiable neuropsychological factor that could be a target for intervention. To date, research into the role of cognitive flexibility in co‐occurring mental health difficulties, particularly among adults, has been hindered by the lack of a validated assessment tool. Recently, the Flexibility Scale‐Self Report has been evaluated and optimal scale structure identified (Hollocks et al. 2023). This identified both a Cognitive Flexibility score and individual indices for Social Flexibility (flexibly engaging elements of typical social interactions through skills such as turn taking and conversation) and Generativity (the ability to spontaneously generate new ideas and problem solve). The development of a self‐report measure of flexibility allows us to explore, from the perspective of autistic adults, whether challenges with cognitive flexibility contribute to associations of perceived stress and social camouflaging with mental health.

## 2 Methods

### 2.1 Participants

Autistic adults were recruited through the Research Match service of the Simons Powering Autism Research (SPARK; The SPARK Consortium 2018) participant registry. Participants completed an online battery of questionnaires, including the Flexibility Scale—Self‐Report (Hollocks et al. 2023), as part of a broader study of autistic adult outcomes. Data were collected during December 2019 and January 2020. Inclusion criteria for the current study were aged ≥ 18 years. Participants with ≥ 20% of missing data from a relevant measure were excluded from analyses. For those with < 20% missing on any given measure, missingness was handled by using the mean item score for the relevant measure.

SPARK participants were “independent” adults, defined by SPARK as persons ≥ 18 years of age who do not have a court‐appointed legal guardian and therefore provide consent for themselves. Based on SPARK's determination of “independent” adult, these participants are unlikely to have a co‐occurring intellectual disability. Further, as part of a detailed medical history collected in the current study, no participant reported a diagnosis of an intellectual disability.

Of 813 participants, 810 (99.6%) self‐disclosed a community‐based professional diagnosis of an autism spectrum condition. Although SPARK does not independently confirm diagnoses, SPARK partners with and recruits from expert autism clinical sites, in part, to increase the likelihood that participants have a professional autism diagnosis (The SPARK Consortium 2018). A separate validation study that examined electronic medical records for 254 SPARK participants, including “independent” adults, confirmed an autism spectrum diagnosis in 98.8% of the sample (Fombonne et al. 2022). Additionally, to characterize the current sample, autistic traits were queried using the 28‐item Autism‐Spectrum Quotient (AQ‐28; Hoekstra et al. 2011), and consistent with the self‐disclosed community‐based autism diagnosis by nearly all of the sample, 94.83% of participants scored above the AQ‐28 cut‐off (> 65) (Table 1).

**TABLE 1:** Participant characteristics.

	*N* = 806
Age, years	
Mean (SD)	40.2 (13.8)
Median (Range)	38.9 (18.2–83.3)
Sex assigned at birth, *n* (%)	
Female	478 (59.3%)
Male	328 (40.7%)
Gender identity, *n* (%)	
Female	416 (51.6%)
Male	316 (39.2%)
Gender non‐conforming	34 (4.2%)
Gender queer	14 (1.7%)
Trans female	4 (0.5%)
Trans male	14 (1.7%)
Another gender identity	6 (0.8%)
Not reported	2 (0.3%)
Ethno‐racial identity, *n* (%)	
Race	
African American or Black	18 (2.2%)
Asian	13 (1.6%)
More than one race	81 (10.1%)
Native American/Native Alaskan	8 (1.0%)
Other	17 (2.1%)
White	666 (82.6%)
Not reported	3 (0.4%)
Ethnicity	
Latinx	67 (8.3%)
Not Latinx	721 (89.5%)
Unknown	12 (1.5%)
Not reported	6 (0.7%)
Educational attainment, *n* (%)	
Less than a bachelor's degree	447 (55.5%)
Bachelor's degree or higher	357 (44.3%)
Not reported	2 (0.2%)
28‐item Autism‐Spectrum Quotient Total Score	
Mean (SD)	84.6 (11.6)
Median (range)	85 (47–112)
AQ ≥ 65, *n* (%)	
Yes	765 (94.9%)
No	41 (5.1%)
Flexibility scale self‐report, cognitive flexibility	
Mean (SD)	22.2 (9.5)
Median (range)	22 (0–45)
Flexibility scale self‐report, social flexibility	
Mean (SD)	7.5 (2.6)
Median (range)	7 (0–15)
GAD‐7	
Mean (SD)	10.1 (6.2)
Median (range)	9 (0–21)
PHQ‐9	
Mean (SD)	10.5 (7.2)
Median (range)	9 (0–27)
PSS	
Mean (SD)	22.9 (7.3)
Median (range)	23 (0–40)
CAT‐Q	
Mean (SD)	110.3 (26.6)
Median (range)	111 (41–174)

*Note:* PHQ‐9 *N* = 803; PSS *N* = 805, CAT‐Q *N* = 789.

**FIGURE 1:**
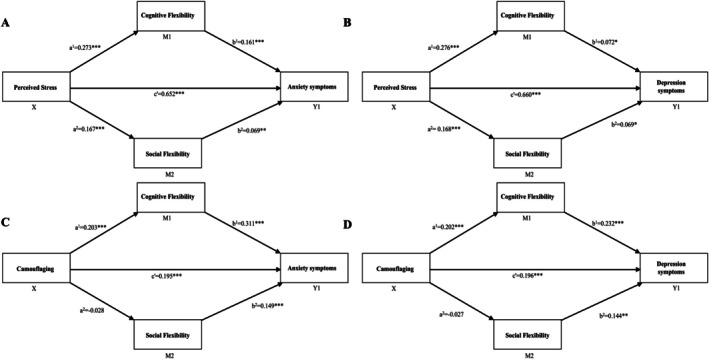
Statistical diagrams of four parallel multiple mediation models of the association between perceived stress and anxiety and depression (A, B) and camouflaging and anxiety and depression (C, D) with cognitive flexibility and social flexibility as mediators. Values correspond to standardized model coefficients (see also Table 3). Covariates for all models were age, sex assigned at birth, and AQ‐28 scores. *p < 0.05; **p < 0.01; ***p < 0.0001. *X* = independent variable; *Y*1 = dependant variable; M = mediator variable; *c*′path represents the direct association between *X* and *Y*; paths *a*_1_, *a*_2_, *b*_1_, *b*_2_, represent coefficients that constitute the indirect effects.

The authors assert that all procedures contributing to this work comply with the ethical standards of the relevant national and institutional committees on human research and with the Helsinki Declaration of 1975, as revised in 2008.

### 2.2 Measures

#### 2.2.1 Cognitive Flexibility

*The Flexibility Scale—Self‐Report* (FS‐SR; Hollocks et al. 2023). The FS‐SR consists of 24 items, with three subscales: (1) Cognitive Flexibility Total; (2) Social Flexibility; and (3) Generativity. The FS‐SR is based on the original Flexibility Scale, which was designed for youth (Strang et al. 2017). Each item is scored on a 4‐point Likert scale (from 0 = No to 3 = Always) with a higher score on each subscale representing lower flexibility. The Flexibility Scale has demonstrated good psychometric properties, including good internal consistency reliability and good construct validity with a strong association with other behavioral measures of executive functioning (i.e., the Behavior Rating Inventory of Executive Function [BRIEF]; Gioia et al. 2000) and a significant association with neuropsychological measures of “switching.” The current version has been adapted for use with autistic adults (Hollocks et al. 2023) and retains adequate internal consistency reliability, with a Cronbach's alpha of 0.88 and 0.71 for the Cognitive Flexibility Score and Social Flexibility subscale, respectively. It is important to highlight that the validation of the adapted FS‐SR scale was partially completed using the current dataset as well as an additional independent dataset. We have included a table of questions included in the FS‐SR in the Supporting Informations. The mean scores for each subscale were used in all analyses.

#### 2.2.2 Camouflaging

*Camouflaging Autistic Traits Questionnaire* (CAT‐Q; Hull et al. 2019). The CAT‐Q is a 25‐item survey that quantifies self‐reported camouflaging during social interactions. Questions are answered on a 7‐point Likert scale (from 1 = Strongly disagree to 7 = Strongly agree), and 7 items are reverse‐scored. Total CAT‐Q scores range from 25 to 175, with higher scores indicating more self‐reported social camouflaging. The CAT‐Q has demonstrated good internal consistency and convergent validity as well as acceptable test–retest reliability (Hull et al. 2019). Cronbach's alpha for the CAT‐Q in the current sample was 0.91. The mean CAT‐Q item score served as the independent variable in all analyses.

#### 2.2.3 Perceived Stress

The Perceived Stress Scale (PSS; Cohen et al. 1983) is one of the most commonly used instruments for the measurement of perceived stress, and has been implemented in samples of autistic adults (McQuaid, Weiss, et al. 2022). The 10‐item version of the PSS was used in the current study. The PSS allows the measurement of a unidimensional scale of global perceived stress. The 10‐item PSS shows good internal consistency in samples of autistic adults (Cronbach's alpha = 0.87; Bishop‐Fitzpatrick et al. 2017) and in prior research involving a sample that overlaps with the one included in the current study (Cronbach's alpha = 0.89; McQuaid, Weiss, et al. 2022). The mean PSS score was used as an independent variable in the relevant analyses.

#### 2.2.4 Depressive Symptoms

The 9‐item Patient Health Questionnaire (PHQ‐9; Kroenke et al. 2001) was used to assess depressive symptomatology and its severity. Participants reported on the presence/frequency of depressive symptoms over the preceding two‐week period using a 4‐point Likert scale (0 = Not at all; 1 = Several days; 2 = More than half the days; 3 = Nearly every day). Responses are summed to generate a total score ranging from 0 to 27. Higher scores are indicative of more severe depressive symptomatology. The PHQ‐9 demonstrates criterion and construct validity and high internal consistency (Cronbach's alpha = 0.86–0.89), and test–retest reliability (intraclass correlation = 0.84; Kroenke et al. 2001). Among sample of autistic adults specifically, the PHQ‐9 has shown excellent internal consistency (Cronbach's alpha = 0.91) and good convergent validity, metrics that were comparable to those of a non‐autistic sample, supporting its utility in samples of autistic adults (Arnold et al. 2020). Mean PHQ‐9 item score served as a dependent variable in analyses.

#### 2.2.5 Anxiety Symptoms

The 7‐item Generalized Anxiety Disorder questionnaire (GAD‐7; Spitzer et al. 2006) was used to assess anxiety. Using a 4‐point Likert scale (0 = Not at all; 1 = Several days; 2 = More than half the days; 3 = Nearly every day), participants reported on the presence/frequency of anxiety during the preceding 2 weeks. A total score is generated by summing responses. Scores range from 0 to 21. Higher scores suggest greater anxious symptomatology. Cronbach's alpha for the GAD‐7 in the current sample was 0.92. Mean GAD‐7 item score served as a dependent variable in analyses.

#### 2.2.6 Autistic Traits

Participants completed the AQ‐28, which queries autistic traits using a 4‐point Likert scale (1 = definitely agree, 4 = definitely disagree; Hoekstra et al. 2011). Total scores range from 28 to 112, with higher scores reflecting greater autistic traits. The AQ‐28 total score has shown good internal consistency in a sample of autistic adults overlapping with the current study (Cronbach's alpha = 0.85; McQuaid et al. 2022). To further characterize the current sample, we determined the number of participants scoring above the AQ‐28 cut‐off (> 65), and we also used the continuous AQ‐28 score as a covariate in our statistical analyses.

### 2.3 Statistical Analysis

Statistical analyses were conducted in R (v.3.6.3), and parallel mediation analyses were implemented using the PROCESS package (Hayes 2022). Mediation modeling is an appropriate statistical approach for various types of data, including data that are cross‐sectional and/or correlational (Hayes 2022, 16–19). Specifically, we follow expert consensus in the use of mediation in cross‐sectional data, including Hayes and Rockwood (2017) and others. These statisticians contend that mediation as a statistical modeling approach is agnostic with respect to the nature of data analyzed, including whether data are correlational versus experimental or cross‐sectional versus longitudinal. Mediation modeling allows us to demonstrate the direct effects of an independent variable on a dependent variable, while simultaneously testing for possible variables (mediating factors), also referred to as indirect associations, which may account for this association. Methods such as mediation can be used in cross‐sectional datasets without concern about implementing the statistical method itself; however, as with all statistical modeling, care must be taken in appropriately framing the interpretation of such results, a point we return to in Section 4.

All variables were explored to ensure normality of distribution and identify any outliers, and no issues with the normality of variables were detected. For all analyses, we used the mean scores from each questionnaire. Bivariate correlations were conducted to explore relationships between the three subscales of the FS‐SR prior to running mediation models, with those not significantly correlated with either anxiety or depression being excluded (see Table 2). Next, four independent parallel multiple mediation models (PROCESS Model 4) were conducted—two with depression as the dependent variable and either perceived stress or CAT‐Q total score as the predictor, and flexibility and social flexibility as the mediators. These were then repeated but with anxiety as the dependent variables. For all models, standardized regression coefficients were generated, and completely standardized indirect effects were produced, and these are reported in all results. Models were considered significantly mediated when the bootstrapped confidence intervals based on 10,000 bootstrap samples did not cross zero.

**TABLE 2:** Correlation matrix showing bivariate associations between variables included in statistical models.

	Cognitive flexibility	Social flexibility	Generativity	Perceived stress	Camouflaging	Depression	Anxiety	Autistic traits	Age
Cognitive flexibility (FS‐SR)	1								
Social flexibility (FS‐SR)	**0.35**	1							
Generativity (FS‐SR)	−0.018	**0.24**	1						
Perceived stress (PSS)	**0.39**	**0.25**	0.011	1					
Camouflaging (CAT‐Q)	**0.34**	**0.098**	**−0.10**	**0.25**	1				
Depression (PHQ‐9)	**0.33**	**0.24**	−0.042	**0.71**	**0.29**	1			
Anxiety (GAD‐7)	**0.42**	**0.27**	−0.065	**0.73**	**0.31**	**0.74**	1		
Autistic traits (AQ‐28)	**0.51**	**0.32**	**0.17**	**0.27**	**0.28**	**0.18**	**0.23**	1	
Age (years)	−0.014	−0.029	−0.054	−0.014	0.051	−0.06	**−0.082**	**0.20**	1

*Note:* Coefficients in bold are significant at *p* < 0.05.

Abbreviations: AQ‐28: autism quotient‐28; CAT‐Q: Camouflaging autistic traits‐questionnaire; FS‐SR: flexibility scale‐self report; GAD‐7: generalized anxiety disorder‐7; PHQ‐9: patient health questionnaire‐9; PSS: perceived stress scale.

**TABLE 3:** Regression standardized coefficients, standard errors, and model summary information for the parallel multiple mediation models depicted in Figure 1.

A. Perceived stress												
*N* = 805		Consequent										
		*M*1 (cognitive flexibility)			*M*2 (social flexibility)					*Y*1 (anxiety)		
Antecedent		Std coeff.	SE	*p*		Std coeff.	SE	*p*		Std coeff.	SE	*p*
X (PSS)	*a*_1_	0.273	0.026	< 0.0001	*a*_2_	0.167	0.020	< 0.0001	*c*	0.652	0.032	< 0.0001
M1 (Cognitive FS‐SR)		—				—			*b*_1_	0.161	0.043	< 0.0001
M2 (Social FS‐SR)		—				—			*b*_2_	0.069	0.054	0.009
C1 (age)		−0.114	0.001	0.0001		−0.101	0.001	0.002		−0.042	0.002	0.095
C2 (sex)		−0.025	0.037	0.397		−0.023	0.029	0.483		0.019	0.044	0.432
C3 (AQ)		0.468	0.047	< 0.0001		0.3344	0.037	< 0.0001		−0.055	0.064	0.063
Constant	i_M1_	−1.013	0.138	< 0.0001	i_M2_	0.083	0.108	0.442	i_Y_	−0.443	0.168	0.009
	*R* ^2^ = 0.357, *F*(4,800) = 110.970, *p* < 0.0001				*R* ^2^ = 0.167, *F*(4,800) = 40.150, *p* < 0.0001				*R* ^2^ = 0.556, *F*(6,798) = 166.342, *p* < 0.0001			

*N* = 803		Consequent										
Antecedent		*M*1 (cognitive flexibility)			*M*2 (social flexibility)					*Y*2 (depression)		
X (PSS)	*a*_1_	0.276	0.026	< 0.0001	*a*_2_	0.168	0.021	< 0.0001	*c*	0.660	0.031	< 0.0001
M1 (Cognitive FS‐SR)		—				—			*b*_1_	0.072	0.041	0.026
M2 (Social FS‐SR)		—				—			*b*_2_	0.069	0.052	0.014
C1 (age)		−0.114	0.001	0.0001		−0.098	0.001	0.003		−0.033	0.002	0.210
C2 (sex)		−0.027	0.037	0.355		−0.022	0.029	0.501		−0.007	0.042	0.788
C3 (AQ)		0.466	0.047	< 0.0001		0.336	0.037	< 0.0001		−0.045	0.061	0.155
constant	i_M1_	−1.005	0.138	< 0.0001	i_M2_	0.073	0.109	0.500	i_Y_	−0.423	0.160	0.009
	*R* ^2^ = 0.356, *F*(4,798) = 110.316, *p* < 0.0001				*R* ^2^ = 0.169, *F*(4,798) = 40.449, *p* < 0.0001				*R* ^2^ = 0.494, *F*(6,796) = 129.394, *p* < 0.0001			

B. Camouflaging												
*N* = 789		Consequent										
		*M*1 (cognitive flexibility)			*M*2 (social flexibility)					*Y*1 (anxiety)		
Antecedent		Std coeff.	SE	*p*		Std coeff.	SE	*p*		Std coeff.	SE	*p*
X (CAT‐Q)	*a*_1_	0.203	0.019	< 0.0001	*a*_2_	−0.028	0.015	0.431	*c*	0.195	0.029	< 0.0001
*M*1 (cognitive FS‐SR)		—				—			*b*_1_	0.311	0.055	< 0.0001
*M*2 (Social FS‐SR)		—				—			*b*_2_	0.149	0.071	< 0.0001
*C*1 (age)		−0.128	0.001	< 0.0001		−0.113	0.001	0.001		−0.060	0.002	0.061
*C*2 (sex)		−0.026	0.039	0.384		0.006	0.030	0.863		0.072	0.058	0.026
*C*3 (AQ)		0.490	0.049	< 0.0001		0.389	0.038	< 0.0001		−0.041	0.085	0.298
Constant	i_M1_	−1.085	0.147	< 0.0001	i_M2_	0.193	0.114	0.091	i_Y_	0.055	0.229	0.810
	*R* ^2^ = 0.327, *F*(4,784) = 95.294, *p* < 0.0001				*R* ^2^ = 0.142, *F*(4,784) = 32.445, *p* < 0.0001				*R* ^2^ = 0.241, *F*(6,782) = 41.412, *p* < 0.0001			

*N* = 786		Consequent										
Antecedent		*M*1 (cognitive flexibility)			*M*2 (social flexibility)					*Y*2 (depression)		
X (CAT‐Q)	*a*_1_	0.202	0.019	< 0.0001	*a*_2_	−0.027	0.015	0.456	*c*	0.196	0.027	< 0.0001
*M*1 (Cognitive FS‐SR)		—				—			*b*_1_	0.232	0.052	< 0.0001
*M*2 (Social FS‐SR)		—				—			*b*_2_	0.144	0.067	0.0001
*C*1 (age)		−0.128	0.001	< 0.0001		−0.113	0.001	0.0008		−0.055	0.002	0.106
*C*2 (sex)		−0.027	0.039	0.367		0.007	0.030	0.830		0.047	0.054	0.164
*C*3 (AQ)		0.488	0.049	< 0.0001		0.390	0.038	< 0.0001		−0.031	0.079	0.448
Constant	i_M1_	−1.079	0.148	< 0.0001	i_M2_	0.1788	0.115	0.119	i_Y_	0.026	0.214	0.902
	*R* ^2^ = 0.325, *F*(4,781) = 93.876, *p* < 0.0001				*R* ^2^ = 0.144, *F*(4,781) = 32.726, *p* < 0.0001				*R* ^2^ = 0.175, *F*(6,779) = 27.601, *p* < 0.0001			

## 3 Results

### 3.1 Descriptive Statistics

The final sample included 806 autistic individuals aged between 18 and 83 years (Mean age = 40.2). Over half of the sample (59.3%) reported female as their sex assigned at birth, with 89.8% identifying as Cisgender. In terms of racial identity, 82.6% of the sample identified as white, with other groups making up a much smaller percentage. Full descriptive statistics and group means for key variables can be found in Table 1.

### 3.2 Bivariate Associations Between Model Variables

A correlation matrix including all key variables was produced (see Table 2) and found that most variables included in the models were significantly correlated. The Cognitive Flexibility score and Social Flexibility subscale score were both significantly correlated with the independent and dependent variables included in the models, hence the decision to run parallel mediation models to look for independent effects. The Generativity subscale was not significantly correlated with these variables and so was not included in any further analyses.

### 3.3 The Meditating Effects of Cognitive and Social Flexibility on the Association Between Perceived Stress and Symptoms of Anxiety and Depression

In the model that included anxiety as the outcome, the total effect was significant (*β* = 0.708; SE = 0.031; 95% CI = 0.801–0.924; *p* < 0.001). There was a significant direct effect of perceived stress on increased anxiety (*β* = 0.652; SE = 0.323; 95% CI = 0.732–0.858; *p* < 0.001). This effect was significantly mediated by both Cognitive Flexibility (completely standardized indirect effect = 0.044; SE = 0.011; 95% bootstrap CIs between 0.024 and 0.066) and social flexibility, which was also found to be a significant mediator of the relationship (completely standardized indirect effect = 0.012; SE = 0.005; bootstrap 95% CIs between 0.002 and 0.023). Age, assigned sex, and autism traits were included as covariates, with increasing age being significantly associated with reductions in anxiety (*p* < 0.01). Contrasts between the mediator effect sizes indicate that they significantly differed in magnitude (completely standardized indirect effect = 0.032; SE = 0.0126; 95% CI = 0.008–0.057; *p* < 0.001) with cognitive flexibility having a larger effect.

When the model was repeated with depression as the outcome, the total effect was significant (*β* = 0.691; SE = 0.029; 95% CI = 0.694–0.809; *p* < 0.001). A significant direct effect from perceived stress was identified (*β* = 0.660; SE = 0.031; 95% CI = 0.657–0.778; *p* < 0.001). This effect was significantly mediated by cognitive flexibility (completely standardized indirect effect = 0.020; SE = 0.0099; bootstrap 95% CIs between 0.0009 and 0.040) and social flexibility was also found to be a significant mediator of the relationship (completely standardized indirect effect = 0.012; SE = 0.006; bootstrap 95% CIs between 0.0016 and 0.0240). Age, assigned sex, and autism traits were included as covariates, but none were found to be significantly associated with depression. The magnitude of mediating effect between cognitive and social flexibility was not significantly different.

### 3.4 The Meditating Effects of Cognitive and Social Flexibility on the Association Between Social Camouflaging and Symptoms of Anxiety and Depression

In the model that included anxiety as the outcome, the total model was significant (*β* = 0.254; SE = 0.030; 95% CI = 0.154–0.271; *p* < 0.001). There was a significant direct effect of social camouflaging on increased anxiety (*β* = 0.195; SE = 0.029; 95% CI = 0.107–0.220; *p* < 0.001). This effect was found to be significantly mediated by cognitive flexibility (completely standardized indirect effect = 0.063; SE = 0.0132; bootstrap 95% CIs between 0.039 and 0.091). However, social flexibility was not found to significantly mediate the direct effect between camouflaging and anxiety (completely standardized indirect effect = 0.004; SE = 0.006; bootstrap 95% CIs between −0.016 and 0.007). Age was found to be a significant covariate in the model (*p* < 0.01).

In the model that included depression as the outcome, the total model was significant (β = 0.2394; SE = 0.027; 95% CI = 0.126–0.233; *p* < 0.001). There was a significant direct effect of social camouflaging on increased depression (*β* = 0.196; SE = 0.027; 95% CI = 0.094–0.200; *p* < 0.001). This direct effect was again found to be significantly mediated by cognitive flexibility.

(completely standardized indirect effect = 0.047; SE = 0.011; bootstrap 95% CIs between 0.026 and 0.071), but not social flexibility (completely standardized indirect effect = −0.004; SE = 0.005; bootstrap 95% CIs between −0.016 and 0.006). Age was found to be a significant covariate in the model (*p* < 0.01).

## 4 Discussion

The aim of this study was to investigate the possible mediating effect of cognitive flexibility on the associations between perceived stress, social camouflaging, and symptoms of anxiety and depression in autistic adults. Prior research has focused on cognitive flexibility as a correlate of co‐occurring mental health conditions experienced by autistic individuals (Hollocks et al. 2022; Lawson et al. 2015; Ozsivadjian et al. 2021; Wallace et al. 2016), but few studies have investigated its potential to contribute to elevated anxiety and depression via its mediating effects on other important risk factors. Due to previous work demonstrating that social flexibility, which focuses specifically on behaviors related to social interaction (turn‐taking, holding conversations etc.), is highly correlated with cognitive flexibility, this was investigated in parallel to identify potential shared or unique effects between the two domains.

Consistent with previous work, we found a significant direct effect of perceived stress (McQuaid, Weiss, et al. 2022) and cognitive flexibility (Hollocks et al. 2022) on elevated symptoms of anxiety and depression. Both cognitive and social flexibility significantly mediated the relationship between perceived stress and greater mental health challenges across both models. In the perceived stress to anxiety model only, cognitive flexibility was found to be a significantly stronger mediator of the relationship when compared to social flexibility. The relationship between the experience of stress and cognitive flexibility is relatively well studied in neurotypical individuals across multiple modalities, including evidence of cortisol‐based reductions in switching ability (Goldfarb et al. 2017), and animal models demonstrating effects of reversal learning and set shifting (Hurtubise and Howland 2017). These relationships have received very little attention to date in the autism literature. Consistent with the current findings, Carter Leno et al. (2022) found that the relationships between stressful childhood experiences and later mental health was moderated by reduced cognitive flexibility. These findings suggest that in the presence of stress, reductions in an individual's ability to think flexibly may increase the likelihood of developing co‐occurring anxiety and depression. This may occur via several different pathways, including an individual's ability to ‘switch’ out of patterns of thought or behavior which are contributing to the development of anxiety and depression or acting to maintain difficulties by presenting a barrier to intervention.

We also found that social flexibility acted as a mediator between perceived stress and both anxiety and depression. Whilst social flexibility is highly correlated with cognitive flexibility (Hollocks et al. 2023), these finding do suggest an independent, but lower magnitude effect. It is likely that social flexibility captures elements of broader constructs such as “social problem‐solving” which have long been identified as being associated with anxiety and depression in neurotypical adults (Romano et al. 2019), and as mediators of response to stress (Kant et al. 1997). Whilst social flexibility was a significant mediator, it was notable that the magnitude of this effect was much smaller when compared to cognitive flexibility when predicting anxiety. Additionally, an examination of the univariate associations indicates a smaller relationship between social (compared to cognitive) flexibility and all key variables. This suggests that in this sample of autistic adults without co‐occurring intellectual disability cognitive flexibility seems the more influential of the processes with regards to associations with both predictors and anxiety/depression. It is also worth considering that given our measure was ‘perceived’ stress, that there could be an element of “all or nothing” (a common cognitive distortion associated with both anxiety and depression) thinking at play which may influences scores. A complementary approach would be to include informant or alternative measurement approaches such as physiological recordings. This is an important methodological issue that could be addressed in future research by the inclusion of informant report or objective measures of stress. Regardless, these findings suggest an important role for cognitive flexibility within this process.

We also found that cognitive, but not social flexibility, was a significant mediator of the relationship between greater social camouflaging behavior and both anxiety and depression. The direct effect between social camouflaging and greater mental health challenges has been found across multiple studies (Hull et al. 2017, 2021; McQuaid et al. 2024). Adding to this established literature, we found that cognitive flexibility contributes to these relationships by mediating the path between social camouflaging and heightened reports of anxiety and depression. It is important to note that as this is a cross‐sectional analysis, we are not able to make cause and effect statements about which of camouflaging or increased mental health challenges comes first. However, recent work has suggested that social camouflaging acts similarly across those with social anxiety (without an autism diagnosis) and can operate as a form of impression management or a safety behavior which may maintain existing anxiety (Lei et al. 2022). Within this context, we would hypothesize that reduced flexibility may contribute to this by impacting an individual's ability to identify when camouflaging behaviors are having a negative impact on mental health and flexibly accessing an alternative response. This is supported by findings that at a neuropsychological level, challenges in cognitive flexibility are in part driven by a pattern of perseverative responding (Crawley et al. 2020; Lage et al. 2023). Importantly, it may be that in some instances, the flexibility challenges could be related to a lack of an available alternative response rather than being driven solely by a neurocognitive process, and the relative contributions of neuropsychological differences and socio‐environmental factors require further investigation. It is likely that the impact of cognitive flexibility differences is not specific to social camouflaging but rather acts as a transdiagnostic risk factor for the development and maintenance of co‐occurring mental health difficulties in autism as a characteristic that tends to be enhanced in this population (Barnes et al. 2025). The finding that Social Flexibility did not mediate the association between social camouflaging and heightened reports of anxiety and depression is likely due to the subscale measuring key aspects of social functioning, which may themselves be the focus of camouflaging behaviors. However, it is important to note that across models, Social Flexibility was significantly associated with anxiety and depression. This likely represents the impact of difficulties with social communication (particularly the elements that overlap with executive functioning) and their impact on mental health.

The finding that reductions in cognitive flexibility mediate the effects of two established but distinct predictors of mental health challenges in autism supports the premise that it could be considered as a transdiagnostic vulnerability factor for the development and maintenance of co‐occurring mental health difficulties. Cognitive flexibility has been consistently linked with both greater internalizing and externalizing symptoms in autistic people (Lei et al. 2022) and suggested to act as a mediator of stability in symptoms over time (Hollocks et al. 2022). However, few studies to date have focused on how cognitive characteristics associated with autism mediate the relationship between other risk exposures or behaviors and mental health. This is particularly important as cognitive flexibility is a potentially modifiable factor that could be a viable intervention target (Kenworthy et al. 2014). However, it is important to note that given this study is cross‐sectional in design and we are unable to ascertain the directionality of effects.

The current study has several strengths, including the use of a large sample of autistic adults with good representation across multiple demographic and background variables, including age, birth‐sex, educational attainment, and gender identity. While on balance this can be considered a strength, an associated limitation is that there are high levels of heterogeneity within the sample. For example, there are subgroups within the current sample for whom we do not have adequate power to run additional analyses, such as those aged 65 and above, where possible aging effects on flexibility may begin to play a role (Giller and Beste 2019). There are also a relatively large proportion of individuals within the sample who identify as gender diverse, and whilst it is beyond the remit of this study, this is a subgroup who would benefit from further research. A key limitation of the current study is that the sample of autistic adults is predominantly White (~84%) and therefore is relatively homogenous in terms of racial diversity. Further efforts should be made to recruit more representative samples and replicate the current findings. In addition, our measurement was reliant on self‐report only, meaning there are potential issues around shared method variance that could be overcome by the inclusion of objective measures or informant reports. An important consideration when interpreting the study results is the factor structure of the FS‐SR. Specifically, the cognitive flexibility subscale is made up of most of the items from the questionnaire, while the social flexibility subscale is composed of seven items. This may indicate a difference in reliability and construct validity of the two subscales and thus may have influenced the current study's findings. Broadly, measurement of cognitive flexibility across studies is inconsistent, and further work on both the validation of behavioral measures (including the FS‐SR) as well as future research that focuses on a multi‐informant and multi‐method approach is vital. Ideally, this could include a longitudinal methodology to enable us to fully understand the complexity of interactions between challenges in flexible thinking and other factors associated with co‐occurring mental health difficulties in autism. It is also important to note that flexibility did not account for the whole relationship between perceived stress, camouflaging, and anxiety/depression. Future research would benefit from exploring a wider range of possible mediating processes.

Despite these limitations there are several clinical implications of these findings. Firstly, supporting autistic individuals to develop strategies to think more flexibility could have an impact on symptoms of anxiety and depression directly, and by supporting autistic individuals to flexibly adapt to different situational demands could reduce the impact of other factors such as environmental and social stressors. One approach that explicitly targets cognitive flexibility and other executive functions, such as planning, is “*Unstuck and On Target!”* (Cannon et al. 2011; Pugliese et al. 2024), which was developed for educators to deliver in classroom settings for autistic students aged 8–11 years and has shown some evidence of effectiveness. Another intervention that shows some promise is Cognitive Remediation Therapy which again targets challenges around flexible thinking along with other executive skills and has been used successfully with those with anorexia nervosa and autistic traits (Dandil et al. 2020). Using either approach, there is potential that supporting autistic people to internalize flexible thinking can shape their resilience and potentially buffer against a range of adversity, and support them to navigate more complex situations by better balancing self‐regulation and goal‐oriented behaviors (Scarpa et al. 2021). Regardless of the approach taken, it is vital to include autistic people in the co‐design of future interventions which may support flexible thinking to ensure acceptability and maximize the chances of developing an effective intervention.

In conclusion, our findings in a relatively large sample of autistic adults suggest that cognitive flexibility plays an important mediating role between the association of both perceived stress and social camouflaging with greater symptoms of both anxiety and depression in autistic adults. This is the first step in trying to elucidate the complex interactions between environmental exposures (stress), behavioral mechanisms related to the development or maintenance (or which may develop in response) of anxiety/depression, and neuropsychological processes. These findings provide us with important routes for further study and will guide thinking on the development of novel and mechanistically informed approaches to clinical care.

## Conflicts of Interest

The authors declare no conflicts of interest.

## Data Availability

The data that support the findings of this study are available from the corresponding author upon reasonable request.
